# Molecular Imaging of Pulmonary Inflammation and Infection

**DOI:** 10.3390/ijms21030894

**Published:** 2020-01-30

**Authors:** Chiara Giraudo, Laura Evangelista, Anna Sara Fraia, Amalia Lupi, Emilio Quaia, Diego Cecchin, Massimiliano Casali

**Affiliations:** 1 Department of Medicine-DIMED,Institute of Radiology, University of Padova, 35100 Padova, Italy; annasara.fraia@gmail.com (A.S.F.); amalialupi88@gmail.com (A.L.); emilio.quaia@unipd.it (E.Q.); 2 Nuclear Medicine Unit, Department of Medicine-DIMED, University of Padova, 35128 Padova, Italy; laura.evangelista@unipd.it (L.E.); cecchin.diego@unipd.it (D.C.); 3 Padova Neuroscience Center (PNC), University of Padova, 35131 Padova, Italy; 4 Azienda Unità Sanitaria Locale–IRCCS di Reggio Emilia, 42121 Reggio Emilia, Italy; massimiliano.casali@ausl.re.it

**Keywords:** lung, inflammation, infection, PET, SPET, PET/CT, PET/MRI

## Abstract

Infectious and inflammatory pulmonary diseases are a leading cause of morbidity and mortality worldwide. Although infrequently used in this setting, molecular imaging may significantly contribute to their diagnosis using techniques like single photon emission tomography (SPET), positron emission tomography (PET) with computed tomography (CT) or magnetic resonance imaging (MRI) with the support of specific or unspecific radiopharmaceutical agents. ^18^F-Fluorodeoxyglucose (^18^F-FDG), mostly applied in oncological imaging, can also detect cells actively involved in infectious and inflammatory conditions, even if with a low specificity. SPET with nonspecific (e.g., ^67^Gallium-citrate (^67^Ga citrate)) and specific tracers (e.g., white blood cells radiolabeled with ^111^Indium-oxine (^111^In) or ^99m^Technetium (^99m^Tc)) showed interesting results for many inflammatory lung diseases. However, ^67^Ga citrate is unfavorable by a radioprotection point of view while radiolabeled white blood cells scan implies complex laboratory settings and labeling procedures. Radiolabeled antibiotics (e.g., ciprofloxacin) have been recently tested, although they seem to be quite unspecific and cause antibiotic resistance. New radiolabeled agents like antimicrobic peptides, binding to bacterial cell membranes, seem very promising. Thus, the aim of this narrative review is to provide a comprehensive overview about techniques, including PET/MRI, and tracers that can guide the clinicians in the appropriate diagnostic pathway of infectious and inflammatory pulmonary diseases.

## 1. Introduction

Infectious and inflammatory pulmonary diseases are a leading cause of morbidity and mortality worldwide. In the United States, lung infections represent the eighth most common cause of death, with an incidence of up to 7 cases per 1000 people per year and are associated with a very high hospitalization rate [1,2]. In Europe, the annual incidence of pneumonia ranges between 1.08 and 1.7 per 1000 people per year [3], with increased mortality rate especially in elderly patients [4].

In addition to pulmonary infections, inflammatory lung diseases are very frequent, such as interstitial lung diseases [5]. Considering the significant burden of such diseases, a prompt diagnosis and early treatment represent the primary goals.

Despite the key role of radiological imaging in detecting and localizing infections or inflammations, since different diseases may cause similar patterns, morphological imaging might be not sufficient to reach the correct diagnosis. Thus, molecular imaging, adding information about the functional activity of the detected lesions, may significantly contribute to both the diagnostic process and the response to treatment. Single photon emission tomography (SPET), and positron emission tomography (PET) or PET/computed tomography (CT) and magnetic resonance imaging (MRI) are very sensitive in detecting the functional/metabolic activity of infectious or inflammatory areas, thus resulting in the useful monitoring of disease activity, mainly after treatment [6].

The aim of this narrative review is to provide a comprehensive overview about techniques and tracers that can guide the clinicians in the appropriate diagnostic pathway.

For the sake of clarity, we divided the argument in three macro areas related to the used tracers: SPET/CT, PET/CT and PET/MRI. Furthermore, two tables, Table 1 and Table 2*,* summarizing the main techniques and tracers that can be used for the assessment of benign lung diseases have been included.

## 2. SPET and SPET/CT

Several clinical studies, demonstrated that in patients with opacities detected at x-ray or CT, ^67^Gallium-citrate (^67^Ga-citrate), ^111^In-oxine (^111^In) or ^99m^Tc hexamethyl propyleneamine-oxime (HMPAO)-labelled autologous leukocytes (white-blood-cell, WBC) scintigraphy may contribute to reach the correct diagnosis of infection or inflammation [7,8].

Despite the successful application of planar scintigraphy or SPET for inflammatory and infectious lung diseases, such techniques are affected by poor spatial resolution [9]. This limit has been partially overcome by the introduction of SPET/CT, allowing not only a precise anatomical localization of the lesions but also a more precise distinction between areas of physiological uptake and infections [10,11,12]. SPET/CT demonstrated a high sensitivity, up to 85% in inflammatory disease, especially when labelled WBC are used [13].

The use of radiolabeled WBC, ^67^Ga-citrate and other SPECT tracers will be discussed in the next three paragraphs.

### 2.1. Radiolabeled WBC

Radiolabelled leukocytes, marked in vitro and reintroduced into the circulation, provide an accurate map of the distribution of labelled WBC within the lung, allowing a robust assessment of pulmonary diseases [14,15]. The characteristics of the uptake depend on chemotaxis, number and types of labelled cells, and the prevalent cellular component involved in the inflammatory response [16].

Since most of the labelled leukocytes are neutrophils and it is well known that bacterial infections cause an immune response mediated by such cells, SPET/CT with radiolabeled WBCs is very sensitive in identifying this type of infections. Nevertheless, it has to be considered that different ranges of accuracy are reported in the literature. For instance, in a series of 13 patients, a full correspondence between the focal uptake at SPET/CT and the clinical and instrumental evidence of infection was achieved [17]. On the contrary, Cook et al. obtained an agreement only in 14 out of 27 cases of pneumonia [18]. Finally, two studies on larger populations showed that ^111^In-WBC SPET/CT has a sensitivity of 94% and a specificity of 64% in detecting pleural and lung infections [14], while it reported a negative predictive value equal to 99% in excluding pulmonary infections in immunocompromised patients [19].

Pulmonary application of SPET and SPET/CT with radiolabeled WBC have been used in animal models for several other diseases. For example, Audi et al. demonstrated in a murine model that ^99m^Tc-HMPAO-WBC uptake occurs 24 h after hyperoxia-induced lung injury and such tracer might be then used as preclinical marker or during follow-up [20].

Despite the current high diagnostic value of SPET/CT and its further promising areas of application, specific challenges have to be considered. First, a recent study involving 20 patients with active bronchiectasis who underwent ^99m^Tc-HMPAO-WBC scan showed that a segmental or lobar uptake might be due labelled leukocytes concentrated in pulmonary bronchiectasis filled of secretions rather than to pneumonia [21]. Second, an accurate interpretation and adequate correlation with clinical findings are necessary. Furthermore, from a technical point of view it is mandatory to distinguish the pathological uptake occurring 4 h after injection of the labelled tracer from the early normal diffuse pulmonary activity (i.e., background activity).

Finally, it has been shown that radiolabeled WBCs have a lower diagnostic value for opportunistic infections (e.g., *Pneumocystis carinii*) and/or mycobacterial infections and sarcoidosis [22,23]. For such diseases, ^67^Ga-citrate has provided better results [24], although in recent years it has been extensively replaced by ^18^F-fluorodeoxyglucose (^18^F-FDG)-PET/CT.

### 2.2. Gallium-67 Citrate

^67^Ga-citrate is an analogue of ferric iron that, after intravenous injection, dissociates from the citrate and binds to the plasmatic transferrin in the circulating blood. Into the extracellular space, the ^67^Ga complex binds with strong avidity to the lactoferrin and the siderophores, which are predominantly released by activated neutrophils and bacteria, thus causing its accumulation in areas affected by infections [25,26]. Nevertheless, considering the intense ^67^Ga-citrate uptake also in presence of agranulocytes, it has to be considered that, similarly to ^18^F-FDG, it is also absorbed by cells with an increased glucose metabolic rate [27]. For this reason, to detect opportunistic pulmonary infections and differentiate infections from Kaposi sarcoma or lymphomas in immunocompromised patients, the use of ^67^Ga-citrate combined with thallium-201 chloride (^201^Tl) has been proposed. In HIV patients with pneumonia due to *Pneumocystis carinii*, a diffuse homogenous pulmonary uptake occurs with ^67^Ga-citrate albeit not with ^201^Tl. On the contrary, Kaposi sarcoma shows pathologic uptake with ^201^Tl but not with ^67^Ga-citrate. Pulmonary lesions due to lymphoma demonstrate metabolic activity with both tracers [28].

Considering lung infections, patients affected by tuberculosis usually show pulmonary parenchymal and nodal pathologic uptake of ^67^Ga-citrate. Although ^18^F-FDG PET is the most widely used molecular imaging technique to assess this infection [29], ^67^Ga-citrate seems to have high sensitivity (83%–100%) in detecting active and inactive lesions, avoiding potential pitfalls due to malignancies, granulomatous disease, and aspergillosis [30,31,32,33,34,35,36,37,38]. The ^67^Ga-citrate uptake is related to activated foamy macrophages, granuloma progression and load of Mycobacterium tuberculosis both in the affected pulmonary sites and in the sputum. Late in 1976, Siemens et al demonstrated a correlation between ^67^Ga-citrate uptake and disease activity in 192 out of 197 patients with active tuberculosis. A progressive decrease of the metabolic activity has been also shown after three to nine months of treatment. Since none of the examined patients with inactive tuberculosis showed pathologic ^67^Ga-citrate uptake, it has been demonstrated that such tracer is accurate in differentiating active disease from fibrosis [30]. However, controversial results have been obtained some years later by Walsh et al. who reported 27% of specificity and 69% of positive predictive value of ^67^Ga-citrate for detecting active foci of tuberculosis [32]. Furthermore, Goswami et al. pointed out that ongoing treatments may reduce the sensitivity of ^67^Ga-citrate in tuberculotic patients [39]. It has also been demonstrated that in active pulmonary tuberculosis, ^67^Ga-citrate uptake correlates with the acid-fast bacilli load in the sputum [40]. According to such evidence, ^67^Ga-citrate has been suggested as an indicator of activated macrophages and bacterial load, guiding the prompt isolation and treatment of patients with active pulmonary disease. Recently, the association of ^67^Ga-citrate with ^111^In, using a novel dual-isotope radiolabeling approach conjugated with vaccines was successfully used in a preclinical animal model to assess the efficacy of a mucosal vaccines for tuberculosis [41].

Rizzato et al. in a multicenter European study [42] demonstrated that ^67^Ga-citrate scintigraphy is more sensitive than chest X-rays for sarcoidosis and that its uptake correlates with the level of angiotensin-converting enzyme (ACE). The typical signs of sarcoidosis at ^67^Ga-citrate imaging are the so-called *lambda* and *panda* signs. These aspects are due to the predominant uptake of the right-sided paratracheal lymph nodes and to the symmetrical uptake in the lacrimal and parotid glands, respectively. Nevertheless, these signs have a poor diagnostic sensitivity in biopsy-proven sarcoidosis patients [43,44,45] and the panda sign may occur in several other diseases (e.g., HIV, lymphomas and Sjogren’s syndrome).

SPET with ^67^Ga-citrate has been applied for inflammatory disorders evolving in pulmonary fibrosis (e.g., idiopathic pulmonary fibrosis (IPF), lymphoid interstitial pneumonitis), for inhalational and occupational pulmonary diseases, (e.g., asbestosis, berylliosis, coal worker pneumoconiosis), collagen vascular diseases (e.g., systemic lupus erythematosus and systemic sclerosis), and other noninfectious inflammatory disease, including pulmonary alveolar proteinosis, eosinophilic pneumonia, Wegener’s granulomatosis, and eosinophilic granuloma [46,47,48,49,50,51,52,53,54,55,56,57,58,59].

Despite the above-mentioned advantages, ^67^Ga-citrate is affected by several limits, such as an adverse dosimetry, long acquisition times, high number of false positives and risk of artifacts. For instance, it requires long intervals (24-72 hours) between the radiopharmaceutical injection and the acquisition [60]. Therefore, its role in clinical practice has progressively reduced, which has created space for the development of new radiopharmaceuticals.

### 2.3. Other SPET/CT Tracers

In addition to the use of radiolabeled WBC and ^67^Ga-citrate, several other molecules have been tested for investigating lung inflammatory and infectious disease by SPET/CT, in humans and in preclinical settings.

In tuberculosis or simil-tuberculotic syndromes, ^99m^Tc-methoxyisobutylisonitrile (MIBI) [61,62] and ^99m^Tc(V)-dimercaptosuccinic acid (DMSA) [63] have been successfully used for pulmonary and extrapulmonary involvement. In 1995, Gulaldi et al. also suggested that a ^99m^Tc-DMSA scanning could represent a valid alternative to ^67^Ga-citrate [64].

Based on the expression of somatostatin receptors in the granulomatous lesions, ^99m^Tc-EDDA-tricine-HYNIC-Tyr3-octreotate has been used as radiotracer for distinguishing between active and inactive tuberculotic lesions [65]. In 2017, Montiero et al. suggested that in pulmonary and extra-pulmonary granulomatous infections SPET/CT with radiotracer as ^99m^Tc-EDDA-HYNIC-TOC or ^111^In-DTPA-octreotide may successfully differentiate active infectious lesions detected on CT from residual scar tissue [66]. As for tuberculosis, radiolabeled analogues of somatostatin, like ^111^In pentetreotide, have been applied for pulmonary and extra thoracic lesions due to sarcoidosis. Kwekkeboom et al. detected a pathologic uptake at SPET in 97% of 46 patients with sarcoid lesions seen on a chest X-ray [67]. Several authors comparing ^67^Ga-citrate with different tracers demonstrated for instance that somatostatin receptor analogues and ^18^F-FDG may even perform better than ^67^Ga-citrate for pulmonary and extrapulmonary lesions [68,69,70,71].

A recent retrospective cross-sectional study evaluated the role of ^99m^Tc-ethambutol SPET/CT in detecting both pulmonary and extra-pulmonary tuberculotic infections, demonstrating, also in pediatric patients, high sensitivity and positive predictive value (both >90%) [72].

Foss et al. using a murine model of pulmonary tuberculosis localized areas of infection by a monoclonal antibody for tissue-bound C3 deposits [73].

In interstitial lung diseases, Zheng et al recently used the collagen-targeting agent ^99m^Tc-CBP1495, in in-vitro and ex-vivo experiments, to assess lung fibrosis [74], being the fibrosis characterized by the pathological accumulation of collagen in the extracellular matrix.

Several preclinical studies addressed the role of different radiolabeled agents selectively binding to antibiotics (e.g., ciprofloxacin, sparfloxacin, enroflaxacin, ceftizoxime, ethambutol, fluconazole) not only for diagnosing infections and inflammatory processes, but also to distinguish one from the other and even differentiate bacterial infection from lesions caused by other pathogens [75,76]. However, some biases related to animal selection and index test, actually limit their transfer from preclinical models to humans [77].

More recently, antimicrobial peptides (AMPs) labeled with isotopes both for SPET (^99m^Tc) and PET imaging (^68^Ga) have grown as more specific agents to localize infections since they specifically bind to bacterial cell membranes. In particular, ubiquicidin (UBI 29-41) has shown encouraging results in human clinical trials [78]. The use of this new category of agents is, to date, mainly limited to the preclinical phase, without strong evidence in the humans and therefore the introduction in the clinical practice seems interesting but far.

Perfusion and ventilation scintigraphy have been used also for chronic obstructive pulmonary disease (COPD) and asthma, both characterized by inflammation and bronchial obstruction. Perfusion scintigraphy is performed using radiolabeled macroaggregate particles, whereas ventilation scintigraphy is conducted by micron-sized, and submicron-sized aerosols (99mTc-labeled DTPA and 99mTc-labeled clusters of carbon particles [Technegas], respectively) or inert radioactive gases (133Xe and 81mKr).

Already in 1997, SPECT with Technegas was successfully applied on healthy volunteers to investigate airway closure [79]. Since then, several studies have explored its use for asthmatic patients. For instance, Farrow et al. demonstrated a significant correlation between severe airway hyperresponsiveness and airway closure at ventilation SPECT/CT [80]. Recently, the same group of researchers examining 14 patients with asthma showed that the ventilation is reduced in areas affected by bronchoconstriction and that the degree of such decrease can be predicted by acinar ventilation heterogeneity [81]. Overall, the role of ventilation scintigraphy for asthma can be considered complementary to functional tests and this technique can be useful for the assessment of the extension of pulmonary involvement, and for monitoring the response to treatment.

Technegas provided very interesting results also for diagnosing and grading the severity of COPD [82]. Using 99mTc-labeled DTPA, the distinction between nonsmoker COPD and asthmatic patients has been achieved in a cohort of 84 patients [Karakavus_83]. In particular, the muco-ciliary clearance, assessed by 99mTc-DTPA in terms of T ½, was significantly higher in patients with asthma than in nonsmoking COPD patients [83].

## 3. PET and PET/CT

PET and PET/CT are mainly used in the oncological field even if it is well known that they play a significant role also for inflammatory and infectious diseases [84,85,86,87,88,89]. Several tracers have been applied for both techniques even if ^18^F-FDG is the most commonly used in clinical practice. The use of ^18^F-FDG and other PET tracers will be discussed in the next two paragraphs.

### 3.1. ^18^F-FDG

**Figure 1:**
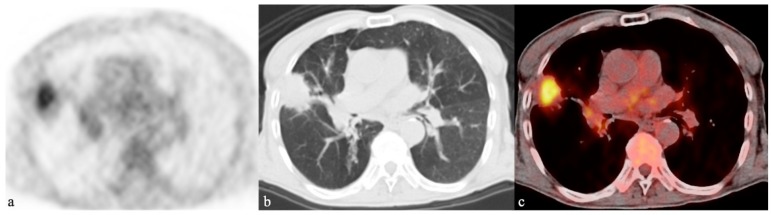
A 79-year-old male affected by pneumonia due to *Pseudomonas aeruginosa* who was examined by ^18^F-FDG-PET/CT. The ^18^F-FDG-PET (**a**), axial CT (**b**), and fused image (**c**) of the ^18^F-FDG-PET/CT scan demonstrate the pulmonary consolidation, with high tracer uptake, in the middle lobe due to the infection.

^18^F-FDG is very sensitive for detecting infections and inflammations because the activated inflammatory cells increase the expression of glucose transporters (GLUT 1, GLUT 3). Moreover, cytokines and grow factors released in inflammatory sites increase the affinity of glucose transporters for the deoxyglucose. ^18^F-FDG uptake is proportional to the cellular metabolic rate [84,89] and binds to the majority of infection and inflammation cells such as neutrophils, lymphocytes, eosinophil and macrophages (Figure 1).

In pulmonary infections ^18^F-FDG-PET/CT has been applied for tuberculosis even if it is characterized by low specificity [90]. Semiquantitative measurements (i.e., standardized uptake values) have not proven to reliably distinguish between infection and pulmonary neoplastic lesions [91,92,93,94].

Several studies reported the use of ^18^F-FDG-PET for monitoring aspergillosis [95] and evaluating the efficacy of chemotherapy in echinococcosis [96]. Indeed, by conventional radiology, parasite viability cannot be assessed, while the detection of metabolic activity with PET may guide the treatment to prevent relapses [97,98]. Bleeker-Rovers et al. demonstrated that the decrease of ^18^F-FDG uptake in PET/CT scan can be applied to determine the success of antifungal therapy for lung abscess due to Candida [99].

In patients affected by sarcoidosis, ^18^F-FDG-PET and PET/CT can play an important role for assessing reversible granuloma, detecting occult diseases, evaluating treatment response and even indicating the most suitable site for biopsy [100,101]. In particular in stage IV that is characterized by fibrosis and mass-like lesions, ^18^F-FDG uptake of reversible granulomas plays a key role in the diagnostic and therapeutic approach [102,103].

To date, only a small number of subjects with either chronic obstructive pulmonary disease or cystic fibrosis have been evaluated with ^18^F-FDG-PET or PET/CT, but the available data suggest that ^18^F-FDG-PET can quantify lung inflammation levels and may thus be a useful biomarker of inflammatory cell activity. ^18^F-FDG uptake may also serve as a biomarker for treatment response to antibiotic therapy, showing a rapid reduction of ^18^F-FDG uptake in patients with acute exacerbations [104,105].

Finally, ^18^F-FDG-PET/CT, combining morphological and anatomical information, is very useful in assessing the extent of extrapulmonary involvement [106].

**Figure 2:**
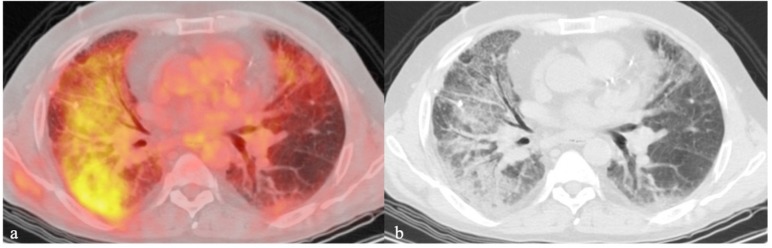
A 60-year-old male with end-stage interstitial lung disease examined by ^18^F-FDG-PET/CT, which demonstrates the high metabolism (**a**) of the affected pulmonary areas (**b**).

The high metabolic rate associated with acute and chronic inflammatory lung diseases, makes ^18^F-FDG especially suitable for assessing and even quantifying the activity of different diffuse or focal inflammatory pulmonary diseases (e.g., smoking-related disease, asthma, cystic fibrosis, organizing pneumonia, IPF, non-specific interstitial pneumonia) [107,108] (Figure 2). ^18^F-FDG uptake can be detected when neutrophils are activated and sequestered within the lungs before their trans-endothelial migration into airways. For this reason, ^18^F-FDG -based techniques can be applied for diagnosing acute lung injury or acute rejection of lung graft [109].

In particular, regarding COPD, a very recent article demonstrated in this group of patients a significant correlation between the metabolic activity of respiratory muscles at ^18^F-FDG-PET/CT and pulmonary function tests [110]. Such evidence unveils a new field of application of this technique.

Furthermore, the oxidative stress, as determinant inflammation factor, may cause pathologic uptake in case of acute rejection or bronchiolitis obliterans after lung transplant as well as for acute lung injury and acute respiratory distress syndrome [111,112].

### 3.2. Other PET/CT Tracers

Besides ^18^F-FDG, other tracers have been tested for pulmonary PET and PET/CT imaging. For instance, ^11^C-choline, ^18^F-fluoroethylcholine (^18^F-FEC), 30-deoxy-30-(^18^F) fluoro-l-thymidine (^18^F-FLT), ^68^Ga-citrate, ^68^Ga-peptides, (^18^F) sodium fluoride (^18^F-NaF), and radiolabeled anti-tuberculosis drugs. In particular, ^11^C- choline and ^18^F-FEC being analogues of lipids are incorporated into the wall of the Mycobacterium. ^11^C-Choline has also been applied to differentiate lung cancer from active tuberculosis but with controversial results [113,114,115].

^18^F-FLT has been tested in a small number of patients affected by sarcoidosis, showing a lower performance than ^18^F-FDG for the identification of extra-cardiac lesions [116].

It has been also demonstrated a correspondence between the increased uptake of ^68^Ga-peptides, such as ^68^Ga-DOTANOC, in areas of peripheral and subpleural abnormalities detected at high-resolution CT in patients with IPF. The association of ^18^F-FDG and ^68^Ga-DOTATOC PET/CT examinations could also provide a combined information about ongoing inflammation and fibroblastic process [117].

^18^F-NaF has been successfully applied in a murine model to detect microcalcifications not detectable at CT in mice with chronic tuberculosis [118], but clinical results are still missing.

Regarding inflammatory diseases, a recent study introduced radiolabeled fluciclatide, an arginine-glycine-aspartic acid peptide, for assessing the angiogenesis and myofibroblast differentiation that characterize the development of pulmonary fibrosis [119].

As several preclinical studies suggested, a radiotracer based on inducible nitric oxide synthase could be useful to study human lung disease but its applicability in clinical settings still needs to be further validated [120,121]. Nevertheless, Huang et al. had already successfully applied one-hour dynamic ^18^F-NOS PET/CT for endotoxin-induced lung inflammation in healthy volunteers [122].

As for SPET, a number of PET studies are ongoing concerning the labelling and initial clinical use of AMPs [123,124,125] mainly labeled with ^68^Ga. Although preliminary AMPs seems very promising in differentiating inflammation and infection.

### 3.3. Magnetic Resonance Imaging (MRI) and PET/MRI

MRI is a well-established technique especially suitable for the assessment of soft tissues and characterized by the extraordinary advantage, among others, of using nonionizing radiofrequency electromagnetic radiations [126,127].

Unfortunately, MRI is not the first choice for pulmonary imaging because of the low proton density of the lungs and the occurrence of movement artifacts due to heartbeats and breathing. Novel imaging techniques like parallel imaging, shared echo-technique, and rotating phase encoding permit to at least partially overcome such limitations. Nevertheless, the lack of clear indications and the necessity of protocols tailored to specific diseases still reduce its application.

Regarding the protocols, a recent review about pediatric chest MRI suggested that fast breath-hold T1 and T2-weighted (T1w and T2w, respectively) images and free breathing steady state free precession sequences provide adequate anatomical and pathological information with a short scan time. Furthermore, it has been shown that respiratory triggered T2-weighted sequences can replace breath-hold sequences in patients with respiratory difficulties or infant, while short tau inversion recovery (STIR) can enhance differences in the signal intensity of pathological areas [126].

Applying the appropriate protocol, despite the above-mentioned limits, MRI demonstrated to be accurate for the assessment of pulmonary infections in animal models as well as in clinical settings [128,129,130,131,132,133,134]. Nowadays, it is well known that areas of high signal intensity on T2w images reflect the presence of intra-alveolar fluid whereas consolidation are hyperintense on T2w images and isointense on T1w images after gadolinium. Abscesses, additionally to the hyperintensity seen on T2w, show restricted diffusion at Diffusion Weighted Imaging (DWI) and rim enhancement; this latter finding is usually absent in case of necrosis. Simple pleural effusion is hyperintense on T2w and hypointense on T1w images, while in case of empyema the pleura appears thickened, shows septa, enhancement and restricted diffusion [129,135,136,137,138].

Some typical CT signs of pulmonary diseases have a direct correspondence on MRI. For instance, Ekinci et al. using T2w balanced fast field echo, T1w turbo spin-echo (TSE), and T2w TSE sequences in the axial and coronal planes, detected the reverse halo sign in immunocompromised patients affected by pneumonia [139]. The same authors, nevertheless highlighted that an accurate diagnosis of tree-in-bud nodules, centrilobular nodules, halo sign and nodules overall, especially if very small, is still challenging at MRI [139].

Ozcan et al. demonstrated that using a radial acquisition method (i.e., BLADE sequence) a higher sensitivity for nodules, ground-glass opacities and consolidation, compared to half-Fourier acquisition single-shot turbo spin-echo, volumetric interpolated breath-hold examination and Fast low angle shot magnetic resonance imaging [140] can be achieved and spectral adiabatic inversion recovery and high resolution isotropic volume examination after contrast are suitable for the evaluation of invasive fungal infection [141]. Moreover, other useful tools such as intravoxel incoherent motion–derived parameters and apparent diffusion coefficient, can be applied for diagnostic purposes, allowing for example the prediction of the treatment response to fungal infection [142].

MRI allows also a distinction between malignant tumors and tuberculomas, having the latter a significantly lower signal intensity on T2w [143,144]. Chung et al. suggested the use of volume-interpolated three-dimensional gradient echo T1w for diagnosing Mycobacterium avium complex pneumonia since it not only allows a robust evaluation of pulmonary nodules and masses but increases the contrast and minimizes the acquisition time and partial volume averaging [145].

Considering other infectious diseases, MRI still has a low specificity for aspergillosis in the early stage, but its diagnostic value is higher in the later stages of the diseases since it well demonstrated the target sign with rim enhancement [146,147].

Considering pulmonary inflammatory diseases, fluid-sensitive sequences are very useful in demonstrating active processes [148] and overall MRI allows the characterization of the extent of the diseases and the differentiation between acute inflammation and fibrosis [149,150,151,152,153].

For instance, in interstitial lung diseases, which are characterized by inflammation and fibrotic remodeling [154], the development of quantitative MRI techniques could help distinguishing between the two components evaluating the differences in relaxation time [155,156,157]. Similarly, it has been shown that ground glass opacities, reticulation and honeycombing have different T2 relaxation values and that T2 values significantly increase with fibrosis. Thus, T2 mapping could play a role in monitoring interstitial lung diseases [158,159]. Moreover, in patients affected by non-specific interstitial pneumonia the T2 relaxation significantly differs between active and stable fibrotic patients [160]. The injection of gadolinium may contribute to the diagnostic process of inflammatory diseases, since the endothelial injury allows the extravascular flow of contrast medium [161].

For the assessment of fibrosis, a gadolinium-based probe targeted to type I collagen (e.g., EP-3533) was successfully applied for diagnosing early disease and monitoring lung fibrosis in an animal model [162,163].

MRI was increasingly applied also in patients with asthma, demonstrating the occurrence of segmental lung edema and being a robust method for monitoring the response to treatment. [164,165,166,167]. Furthermore, it has been shown that hyperpolarized gases allow the evaluation of functional changes in distal small airways of asthmatic patients. In particular, using an isotope of helium (i.e., 3HE), hyperpolarized through a circularly polarized laser light and administered as inhalation contrast media, lung airspaces can be assessed, and ventilation defects diagnosed since they appear as signal voids [168,169,170,171,172,173,174].

**Figure 3:**
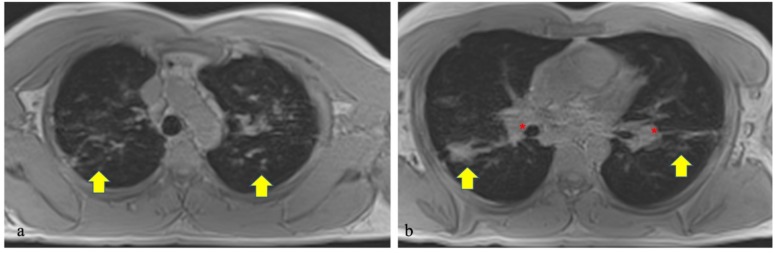
Short echo-time of the lungs showing multiple pulmonary nodules and enlarged hilar lymph nodes (respectively, yellow arrows and red asterisks in (**a**) and (**b**)) in a 40-year-old male patient affected by sarcoidosis.

In specific clinical conditions like cystic fibrosis, MRI can be even preferable than CT, despite the lack of details, given the risk associated with long term radiation exposure associated with multiple CT scans. MRI has already proven to be reliable in the evaluation of structural changes like bronchiectasis, wall thickening, mucus plugging, and infiltrates [126,171,172,173] and novel sequences such as ultrashort or zero echo time and multiparametric-functional imaging are expected to provide additional information (Figure 3). Furthermore, scores based on Diffusion weighted imaging demonstrated to strongly correlate with the severity of the disease and the amount of mucus [174] as well as of being associated with symptoms scores, spirometry measurements and inflammation during disease’s exacerbation [164,175,176]. Hyperpolarized gases such as 129Xe showed inhomogeneous ventilation patterns with areas of lacking ventilation in such patients [177].

**Figure 4:**
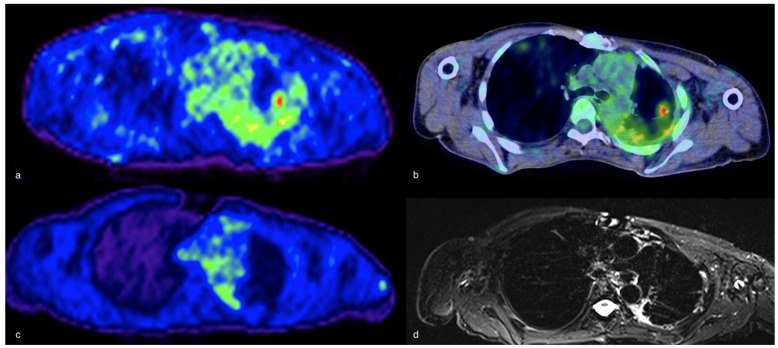
^18^F-FDG-PET (**a**) and fused axial ^18^F-FDG-PET/CT (**b**) demonstrating a pulmonary infection with high metabolic activity in the left upper lobe which then decreased after treatment with antibiotics at ^18^F-FDG-PET/MR (fused axial image in (**c**) and axial STIR in (**d**).

According to the recent above-mentioned evidence, MRI is progressively becoming a valid radiation-free alternative that will increasingly be applied in the next years, taking full advantage of all technological progresses and of the expanding use of hybrid techniques, such as PET/MRI [129,138,178,179,180,181,182,183,184] (Figure 4).

Indeed, PET/MRI, providing simultaneous metabolic and functional information, is a robust technique with a broad spectrum of applications especially in the oncological field. Also for pulmonary imaging, it has been successfully applied for the assessment of primary and metastatic lesions [185,186,187,188,189,190]. Regarding infectious diseases, in a recent study, ^18^F-FDG-PET/MR showed a similar performance than PET/CT in detecting pulmonary lesions due to tuberculosis [191]. Nevertheless, further research, including multicenter research trials, is needed for establishing the role of this diagnostic tool for inflammatory and infectious lung diseases.

## 4. Future Perspectives

The upcoming technical progress in molecular imaging and the transposition into clinical settings of the knowledge gained in experimental preclinical studies are expected to change our perspectives and introduce new diagnostic tools for pulmonary imaging [127,180].

For instance, we may expect the application of ^19^F markers, such as perfluorcarbon compounds, which allow to track stem and immune cells. Indeed, in experimental disease models, it has been demonstrated that taking advantage of the negligible ^19^F background signal, inflammation can be assessed [192,193].

Furthermore, fluorescence and bioluminescence, using the optical properties of tissues originated from biochemical reactions and biological processes, through the use of fluorochromes, reporter genes and optical contrast agents, will favor even greater versatility in nuclear imaging, although to date clinical applications for optical imaging are still missing [194,195].

## 5. Conclusions

In conclusion, molecular imaging could potentially play a key role for inflammatory and infectious pulmonary diseases. SPET and SPET/CT using radiolabeled WBC allow for the characterization of pneumonia, while tuberculosis and inflammatory disease have been historically assessed using ^67^Ga-citrate. However, for radioprotection reasons and worldwide availability, ^67^Ga-citrate will be presumably abandoned. ^18^F-FDG-PET/CT, providing information about the glucose metabolism, is highly reliable to detect active lesions for several types of lung diseases like tuberculosis, sarcoidosis and inflammatory disease in general, but the low specificity represents an important limitation. We could hypothesize that radiolabeled (probably with ^68^Ga) AMPs, specifically binding to bacterial cell membranes could, in the future, be used as a second line after ^18^F-FDG to increase specificity of the diagnostic process. In addition, we expect an increasing application of MRI and PET/MRI in this setting.

## Figures and Tables

**Table 1:** Tracers, techniques and targets.

Tracer	Technique	Target
18F-FDG	PET	Neutrophils, lymphocytes, eosinophils and macrophages
67Ga/68Ga citrate	SPECT/PET	Neutrophils and leukocytes
99mTc-leukocites	SPECT	Leukocytes
111In/68Ga-Somatostatic receptors	SPECT/PET	Lymphocytes, monocytes and macrophages (also fibroblasts)
11C-PK11195	PET	Monocities and neutrophils

FDG: fluorodeoxyglucose; PET: positron emission tomography; SPECT: single photon emission tomography;Ga: Gallium; In: Indium; 11C: 11Choline.

**Table 2:** Clinical indications and main radiopharmaceutical agents for the assessment of various benign lung diseases.

Tracer	Disease	Clinical Indication
Radiolabeled WBC	Pneumonia	-
	Pleural and lung infections	Diagnosis
	Mycobacterial infections	Diagnosis
^67^Ga-citrate	Pulmonary infections	-
	Pneumonia*	-
	Tuberculosis	Diagnosis, evaluation of response to therapy, active vs. nonactive disease
	Sarcoidosis	Diagnosis
	Pulmonary fibrosis	-
FDG	Tuberculosis	-
	Sarcoidosis and extrapulmonary involvement	Diagnosis, evaluation of response to therapy, biopsy guide

WBC=white blood cell; FDG=fluorodeoxyglucose; *especially in immunocompromised patients; Ga: Gallium.
